# Adherence to the Mediterranean diet is inversely associated with hematologic inflammatory indices in adults seeking a weight loss dietary program: a cross-sectional study

**DOI:** 10.1007/s00394-026-04075-8

**Published:** 2026-08-06

**Authors:** Francesca Menichetti, Silvia Gallosti, Ramona De Amicis, Stefano Ravasenghi, Giovanni Fiorillo, Franca Criscuoli, Andrea Foppiani, Simona Bertoli, Alberto Battezzati, Alessandro Leone

**Affiliations:** 1 International Center for the Assessment of Nutritional Status and development of Dietary Intervention Strategies (ICANS-DIS), Department of Food, Environmental and Nutritional Sciences (DeFENS), University of Milan, Milan, Italy; 2 Department of Endocrine and Metabolic Diseases, Obesity Unit and Laboratory of Nutrition and Obesity Research, Istituto Auxologico Italiano, IRCCS, Milan, Italy; 3 Department of Endocrine and Metabolic Medicine, Clinical Nutrition Unit, Istituto Auxologico Italiano, IRCCS, Milan, Italy

**Keywords:** Mediterranean diet, Low-grade inflammation, Inflammatory indices, Obesity

## Abstract

**Background:**

Low-grade systemic inflammation is a hallmark of excess adiposity and contributes to cardiometabolic risk. Adherence to the Mediterranean diet has been associated with reduced inflammation. However, evidence linking Mediterranean diet to composite inflammatory indices derived from routine blood tests is limited, particularly in individuals seeking weight loss interventions.

**Methods:**

In this cross-sectional study, 1,738 adults requesting a weight loss dietary program underwent anthropometric assessment, body composition evaluation, and fasting blood sampling. Adherence to the Mediterranean diet was assessed using the 14-item Mediterranean Diet Adherence Screener (MEDAS). Hematologic inflammatory indices, including neutrophil-to-lymphocyte ratio (NLR), platelet-to-lymphocyte ratio (PLR), systemic immune-inflammation index (SII), lymphocyte-to-monocyte ratio (LMR), systemic inflammation response index (SIRI), and monocyte-to-HDL ratio (MHR), were calculated.

**Results:**

After adjustments for sex, age, BMI, body fat percentage, metabolic syndrome, smoking status, physical activity, education level, and marital status, each 1-point increase in MEDAS score was associated with lower NLR (β = − 0.065; 95% CI: − 0.102, − 0.028, *p* = 0.001), PLR (β = − 2.660; 95% CI: − 4.570, − 0.749, *p* = 0.006), SII (β = − 21.233; 95% CI: − 31.520, − 10.947, *p* < 0.001), SIRI (β = − 0.046; 95% CI: − 0.068, − 0.024, *p* < 0.001), and MHR (β = − 0.0002; 95% CI: − 0.0003, − 0.0001, *p* = 0.003), and with higher LMR (β = 0.078; 95% CI: 0.038, 0.117, *p* < 0.001), all reflecting a shift toward a more favorable inflammatory profile.

**Conclusion:**

Greater adherence to the Mediterranean diet was associated with a more favorable inflammatory profile. These results support the role of the Mediterranean diet in modulating low-grade systemic inflammation in clinical nutrition settings.

## Introduction

Low-grade chronic inflammation is a condition closely associated with excess body fat and is characterized by alterations in circulating immune cells and an increased release of pro-inflammatory mediators [1–4]. This state predisposes individuals with excess adiposity to a higher risk of developing obesity-related comorbidities, such as insulin resistance, dyslipidemia, diabetes, and cardiovascular disease [5, 6].

It is well established that diet can modulate the inflammatory response [7]. In recent years, interest in the role of different dietary patterns in regulating inflammation has grown considerably. Among these patterns, the Mediterranean diet is one of the most extensively studied due to its protective effects on health, including reduced risk of type 2 diabetes, cardiovascular disease, and all-cause mortality [8]. This dietary pattern is characterized by a high intake of plant-based foods, moderate consumption of fish and wine, limited intake of red meat and refined products, and the use of extra-virgin olive oil as the primary source of fat [9].

A growing body of evidence has investigated the potential anti-inflammatory effects of the Mediterranean diet, showing that higher adherence to this dietary pattern is associated with reduced levels of C-reactive protein and interleukins (IL-1β, IL-6, and IL-17) [10, 11]. However, many of the inflammatory biomarkers frequently assessed in research, such as cytokines and specific proteins, are not routinely measured in clinical settings. For this reason, inflammatory indices derived from complete blood counts have gained increasing attention as simple, accessible, and cost-effective markers of systemic inflammation, particularly in large-scale epidemiological studies [12]. These include the neutrophil-to-lymphocyte ratio (NLR), platelet-to-lymphocyte ratio (PLR), systemic immune-inflammation index (SII), lymphocyte-to-monocyte ratio (LMR), systemic inflammation response index (SIRI), and monocyte-to-HDL ratio (MHR). These indices integrate blood cell counts and lipid parameters readily available from standard laboratory tests and have proven useful in describing the degree of systemic inflammation in several cardiometabolic conditions, including metabolic syndrome, type 2 diabetes, and non-alcoholic fatty liver disease [13–18].

Despite the growing interest in these indices, evidence regarding their association with adherence to the Mediterranean diet remains limited, particularly in adults participating in weight-loss programs. In such individuals, who commonly present with low-grade chronic inflammation and increased cardiometabolic risk, understanding the impact of diet quality on inflammatory blood markers could have important clinical implications.

In light of these considerations, this study aims to explore the association between adherence to the Mediterranean diet and a broad panel of blood-derived inflammatory markers in adults seeking a weight-loss dietary program.

## Materials and methods

### Study design and participant selection

This retrospective cross-sectional analysis was based on clinical data collected from adults who attended the International Center for the Assessment of Nutritional Status (ICANS) between January 2016 and December 2020. Eligibility was restricted to adults aged ≥ 18 years who did not present with hepatitis B or C virus infection, acute infection, liver cirrhosis, neurological disorders, gastrointestinal diseases, heart failure, renal failure, pulmonary insufficiency, or ongoing treatment with drugs known to induce lipodystrophy. A total of 2524 individuals met these eligibility criteria. Participants with missing information on variables included in the analyses (*n* = 486), as well as those with diabetes, cardiovascular disease, or a history of cancer within the previous five years (*n* = 300), were excluded. The final analytical sample consisted of 1,738 adults.

At enrollment, participants underwent a comprehensive medical interview covering personal and family medical history, current medications, smoking status, and physical activity habits. Blood pressure was measured by a physician in accordance with the Joint National Committee 7 guidelines [19]. As part of the baseline assessment, anthropometric measurements, fasting blood sampling, and dietary evaluation through a validated Mediterranean Diet questionnaire were performed for all participants. All participants provided written informed consent, and the study protocol was approved by the Ethics Committee of the University of Milan (Protocol No. 23/2016), in line with the Helsinki Declaration.

### Anthropometric measurements

All anthropometric measurements were performed by a trained anthropometrist following international standard procedures [20]. All operators received specific training to ensure the reproducibility of measurements and to minimize inter-observer variability. Participants wore only underwear and socks during measurements. Body weight was recorded using a column scale with a precision of 0.1 kg, while height was measured with a stadiometer to the nearest 0.5 cm. Body Mass Index (BMI) was calculated as weight (kg) divided by height squared (m²) and categorized according to WHO guidelines: normal weight (18.5–24.9 kg/m²), overweight (25–29.9 kg/m²), or obesity (≥ 30 kg/m²). Waist circumference was measured at the midpoint between the lowest rib and the iliac crest using a non-elastic tape. Skinfold thickness was measured at four sites—triceps, biceps, subscapular, and suprailiac—using a caliper with 0.2 mm precision. Each site was measured three times and the average was recorded. Measurements were performed on the non-dominant side. At our center, the intra-observer variation for skinfold measurements ranges between 2.5% and 2.9% [21]. Body density was estimated according to Durnin and Womersley equations [22], taking age and sex into account, and body fat percentage was calculated using Siri’s formula [23]: Body fat % = (4.95 / ρ − 4.50) × 100.

### Assessment of Mediterranean diet adherence

Adherence to the Mediterranean diet was assessed using the self-administered 14-item MEDAS questionnaire, validated in the PREMIDED trial [24]. Each item evaluates the consumption frequency of foods characteristic of Mediterranean versus Western diets. Participants received one point for each item meeting the Mediterranean criterion, resulting in a total score ranging from 0 to 14, with higher scores indicating greater adherence to the Mediterranean diet [25]. Briefly, 1-point was attributed for each of the following: (1) olive oil as the main cooking fat; (2) olive oil ≥ 4 tablespoons/day; (3) vegetables ≥ 2 servings/day; (4) fruit ≥ 3 servings/day; (5) red or processed meat < 1 serving/day; (6) butter or cream or margarine < 1/day; (7) sugar-sweetened beverages < 1/day; (8) wine ≥ 3 glasses/week; (9) legumes ≥ 3 servings/week; (10) fish/seafood ≥ 3 servings/week; (11) commercial sweets and confectionery < 3/week; (12) nuts ≥ 1/week; (13) white more than red meats (yes) and; (14) use of soffritto ≥ 2/week [26, 27].

### Blood sampling and inflammatory indices

Fasting blood samples were collected between 8:30 and 9:30 a.m. Complete blood counts were obtained to measure neutrophils, lymphocytes, monocytes, and platelets. Fasting glucose, HDL-cholesterol and triglycerides were measured using enzymatic methods (Cobas Integra 400 Plus; Roche Diagnostics). Several systemic inflammatory indices were calculated according the following formulas:

$$\:NLR=\:\frac{\mathrm{n}\mathrm{e}\mathrm{u}\mathrm{t}\mathrm{r}\mathrm{o}\mathrm{p}\mathrm{h}\mathrm{i}\mathrm{l}\:\mathrm{c}\mathrm{o}\mathrm{u}\mathrm{n}\mathrm{t}\:}{\mathrm{l}\mathrm{y}\mathrm{m}\mathrm{p}\mathrm{h}\mathrm{o}\mathrm{c}\mathrm{y}\mathrm{t}\mathrm{e}\:\mathrm{c}\mathrm{o}\mathrm{u}\mathrm{n}\mathrm{t}}$$

$$\:PLR=\:\frac{\mathrm{p}\mathrm{l}\mathrm{a}\mathrm{t}\mathrm{e}\mathrm{l}\mathrm{e}\mathrm{t}\:\mathrm{c}\mathrm{o}\mathrm{u}\mathrm{n}\mathrm{t}}{\mathrm{l}\mathrm{y}\mathrm{m}\mathrm{p}\mathrm{h}\mathrm{o}\mathrm{c}\mathrm{y}\mathrm{t}\mathrm{e}\:\mathrm{c}\mathrm{o}\mathrm{u}\mathrm{n}\mathrm{t}}$$

$$\:SII=\:\frac{\mathrm{p}\mathrm{l}\mathrm{a}\mathrm{t}\mathrm{e}\mathrm{l}\mathrm{e}\mathrm{t}\:\mathrm{c}\mathrm{o}\mathrm{u}\mathrm{n}\mathrm{t}\:\times\:\:\mathrm{n}\mathrm{e}\mathrm{u}\mathrm{t}\mathrm{r}\mathrm{o}\mathrm{p}\mathrm{h}\mathrm{i}\mathrm{l}\:\mathrm{c}\mathrm{o}\mathrm{u}\mathrm{n}\mathrm{t}}{\mathrm{l}\mathrm{y}\mathrm{m}\mathrm{p}\mathrm{h}\mathrm{o}\mathrm{c}\mathrm{y}\mathrm{t}\mathrm{e}\:\mathrm{c}\mathrm{o}\mathrm{u}\mathrm{n}\mathrm{t}}$$

$$\:LMR=\:\frac{\mathrm{l}\mathrm{y}\mathrm{m}\mathrm{p}\mathrm{h}\mathrm{o}\mathrm{c}\mathrm{y}\mathrm{t}\mathrm{e}\:\mathrm{c}\mathrm{o}\mathrm{u}\mathrm{n}\mathrm{t}}{\mathrm{m}\mathrm{o}\mathrm{n}\mathrm{o}\mathrm{c}\mathrm{y}\mathrm{t}\mathrm{e}\:\mathrm{c}\mathrm{o}\mathrm{u}\mathrm{n}\mathrm{t}}$$

$$\:SIRI=\:\frac{\mathrm{n}\mathrm{e}\mathrm{u}\mathrm{t}\mathrm{r}\mathrm{o}\mathrm{p}\mathrm{h}\mathrm{i}\mathrm{l}\:\mathrm{c}\mathrm{o}\mathrm{u}\mathrm{n}\mathrm{t}\:\times\:\:\mathrm{m}\mathrm{o}\mathrm{n}\mathrm{o}\mathrm{c}\mathrm{y}\mathrm{t}\mathrm{e}\:\mathrm{c}\mathrm{o}\mathrm{u}\mathrm{n}\mathrm{t}}{\mathrm{l}\mathrm{y}\mathrm{m}\mathrm{p}\mathrm{h}\mathrm{o}\mathrm{c}\mathrm{y}\mathrm{t}\mathrm{e}\:\mathrm{c}\mathrm{o}\mathrm{u}\mathrm{n}\mathrm{t}}$$

$$\:MHR=\:\frac{\mathrm{m}\mathrm{o}\mathrm{n}\mathrm{o}\mathrm{c}\mathrm{y}\mathrm{t}\mathrm{e}\:\mathrm{c}\mathrm{o}\mathrm{u}\mathrm{n}\mathrm{t}}{\mathrm{H}\mathrm{D}\mathrm{L}-\mathrm{C}\:(\mathrm{m}\mathrm{g}/\mathrm{d}\mathrm{l})}$$

Metabolic syndrome was defined according to the harmonized international criteria [28]. A large waist circumference was classified as ≥ 102 cm in men and ≥ 88 cm in women. Low HDL-cholesterol was defined as < 40 mg/dl in men and < 50 mg/dl in women. Elevated triglycerides were defined as ≥ 150 mg/dl or current use of triglyceride-lowering medication. High blood pressure was defined as systolic blood pressure ≥ 130 mm Hg, diastolic blood pressure ≥ 85 mm Hg, or use of antihypertensive drugs. Elevated fasting glucose was defined as ≥ 100 mg/dl or treatment with glucose-lowering medication. Metabolic syndrome was diagnosed when at least three of these components were present.

### Statistical analysis

Continuous variables are described as median and interquartile range (25^th^ − 75^th^ percentiles, IQR) due to the non-normal distribution of several variables, as assessed using the Kolmogorov–Smirnov test. Categorical variables are expressed as absolute frequencies and percentages. The association between adherence to the Mediterranean diet, expressed as the MEDAS score, and hematologic inflammatory indices was investigated using linear regression models. Regression analyses were adjusted a priori for potential confounders including sex (binary), age (continuous), body mass index (continuous), body fat percentage (continuous), metabolic syndrome (binary), smoking status (categorical: never, former, current), physical activity (binary: <2 vs. ≥ 2 h/week), education level (categorical: elementary or middle school, high school, master degree or higher), and marital status (categorical: not married, married, divorced or widower). Model assumptions were carefully evaluated. Multicollinearity among predictors was assessed using the Variance Inflation Factor (VIF), with all values remaining below the commonly accepted threshold (< 5), indicating no relevant collinearity. The linearity assumption between continuous covariates and inflammatory outcomes was explored using multivariable fractional polynomials. Homoscedasticity was examined through visual inspection of residual-versus-fitted plots and formally tested using the Breusch–Pagan test. In the presence of heteroscedasticity, robust confidence intervals were applied to ensure valid inference. All statistical tests were two-sided, and a p-value < 0.05 was considered statistically significant. Statistical analyses were conducted using STATA software, version 18.5 (StataCorp LP, College Station, TX, USA).

## Results

The study included 1,738 participants, of whom 72.4% were women and 27.6% were men. The median age of the study population was 46 years (IQR: 36–54), while the median BMI was 27.6 kg/m² (IQR: 24.9–30.7). Overall, 26.4% of participants were classified as normal weight, 44.1% as overweight, and 29.5% as presenting with obesity. Tables 1 and 2 report the main characteristics of the recruited sample.

**Table 1:** Characteristics of patients

	Terciles of adherence to the Mediterranean diet											
	1^st^ tercile			2^nd^ tercile			3^rd^ tercile			Total		
	Median	P25	P75	Median	P25	P75	Median	P25	P75	Median	P25	P75
Age (years)	44	35	52	46	37	54	48	39	56	46	36	54
BMI (kg/m^2^)	27.9	25.3	31.0	27.4	24.3	30.3	27.1	24.6	30.4	27.6	24.9	30.7
Body fat (%)	36.2	31.1	40.0	35.7	31.4	39.4	36.4	30.8	40.2	36.1	31.1	40.0
Waist circumference (cm)	94.5	86.7	105.0	92.6	83.5	102.5	93.0	85.0	101.7	94.0	85.5	103.2
Total cholesterol (mg/dl)	202	176	229	206	178	233	202	177	232	203	177	231
LDL cholesterol (mg/dl)	133	109	160	137	112	162	134	110	158	134	110	160
HDL (mg/dl)	58	49	70	62	51	72	63	52	72	60	50	71
Triglycerides (mg/dl)	87	65	132	84	61	115	81	60	116	85	62	121
Glucose (mg/dl)	95	89	101	95	90	102	95	90	102	95	90	101
Systolic blood pressure (mm Hg)	120	110	130	120	110	130	120	110	130	120	110	130
Diastolic blood pressure (mm Hg)	80	70	82	76	70	80	75	70	80	78	70	80
Neutrophil count (10^3/microl)	3.40	2.80	4.20	3.30	2.70	4.00	3.10	2.50	3.80	3.30	2.70	4.00
Lymphocyte count (10^3/microl)	1.80	1.50	2.20	1.80	1.40	2.20	1.70	1.40	2.20	1.80	1.47	2.20
Monocyte count (10^3/microl)	0.50	0.40	0.60	0.50	0.40	0.60	0.40	0.40	0.60	0.50	0.40	0.60
Platelet count (10^3/microl)	244	212	286	247	212	279	242	208	273	244	210	280
NLR	1.9	1.5	2.7	1.8	1.4	2.5	1.8	1.4	2.3	1.9	1.4	2.5
PLR	137	109	175	137	110	169	137	109	168	137	109	171
SII	476	345	668	447	324	604	428	315	577	456	328	619
LMR	3.7	2.8	4.6	4.0	3.0	5.0	4.0	3.0	5.0	3.8	3.0	4.8
SIRI	1.0	0.7	1.3	0.8	0.6	1.2	0.8	0.6	1.1	0.9	0.6	1.3
MHR	0.008	0.006	0.012	0.007	0.005	0.011	0.007	0.005	0.010	0.008	0.006	0.011
Mediterranean score	5	5	6	7	7	7	8	8	9	7	6	8

Abbreviations: P25, 25^th^ percentile; P75, 75^th^ percentile; NLR, neutrophil-to-lymphocyte ratio; PLR, platelet-to-lymphocyte ratio; SII, systemic immune-inflammation index; LMR, lymphocyte-to-monocyte ratio; SIRI, systemic inflammation response index; MHR, monocyte-to-HDL ratio

**Table 2:** Socio-demographic, nutritional and metabolic characteristics of recruited patients

	Terciles of adherence to the Mediterranean diet							
	1^st^ tercile		2^nd^ tercile		3^rd^ tercile		Total	
	N	%	N	%	N	%	N	%
Sex								
Female	557	69.1	295	74.9	406	75.5	1258	72.4
Male	249	30.9	99	25.1	132	24.5	480	27.6
BMI classes								
Normal weight	183	22.7	119	30.2	156	29	458	26.4
Overweight	366	45.4	163	41.4	238	44.2	767	44.1
Obesity	257	31.9	112	28.4	144	26.8	513	29.5
Physically active (≥ 2 h/week)	443	55	237	60.2	339	63	1019	58.6
Smoking								
Not smoker	434	53.8	206	52.3	296	55	936	53.9
Ex smoker	99	12.3	57	14.5	74	13.8	230	13.2
Smoker	273	33.9	131	33.2	168	31.2	572	32.9
Education								
Elementary or middle school	61	7.6	24	6.1	35	6.5	120	6.9
High school	347	43.1	171	43.4	218	40.5	736	42.3
Master degree or higher	398	49.4	199	50.5	285	53	882	50.7
Marital status								
Not married	373	46.3	172	43.7	207	38.5	752	43.3
Married	376	46.7	195	49.5	297	55.2	868	49.9
Divorced or widower	57	7.1	27	6.9	34	6.3	118	6.8
High triglycerides (≥ 150 mg/dl or treatment)	138	17.1	49	12.4	71	13.3	258	14.9
Low HDL (< 50 F/40 M mg/dl or treatment)	147	18.2	57	14.5	71	13.2	275	15.8
High blood pressure (≥ 130/85 mm Hg or treatment)	292	39.4	125	34.5	172	36.9	589	37.5
Impaired fasting glucose (≥ 100 mg/dl or treatment)	246	30.6	138	35	179	33.3	563	32.5
Metabolic syndrome	202	25.1	86	21.8	109	20.3	397	22.8

Table 3 presents the results of linear regression analyses evaluating the association between MEDAS score and hematologic inflammatory indices. In crude models, higher MEDAS scores were significantly associated with lower NLR, PLR, SII, SIRI and MHR values, and with higher LMR values. These associations remained substantially unchanged after adjustment for sex, age, and BMI (Model 1). In the fully adjusted model (Model 2), which additionally accounted for body fat percentage, metabolic syndrome, smoking status, physical activity, education level, and marital status, each 1-point increase in the MEDAS score was independently associated with lower NLR (β = −0.065; 95% CI: −0.102, − 0.028; *p* = 0.001), PLR (β = −2.660; 95% CI: −4.570, − 0.749; *p* = 0.006), SII (β = −21.233; 95% CI: −31.520, − 10.947; *p* < 0.001), SIRI (β = −0.046; 95% CI: −0.068, − 0.024; *p* < 0.001) and MHR (β = −0.0002; 95% CI: −0.0003, − 0.0001; *p* = 0.003). Moreover, a 1-point increment in MEDAS score was associated with a significant increase in LMR (β = 0.078; 95% CI: 0.038, 0.117; *p* < 0.001).

**Table 3:** Association of Mediterranean diet score with inflammatory indices

	NLR	PLR	SII	LMR	SIRI	MHR
Crude model	-0.065 [-0.098,-0.033]^***^	-1.792 [-3.523,-0.061]^*^	-22.265 [-31.196,-13.334]^***^	0.079 [0.039,0.119]^***^	-0.053 [-0.073,-0.033]^***^	-0.0003 [-0.0005,-0.0002]^***^
M1	-0.066 [-0.101,-0.030]^***^	-2.613 [-4.460,-0.766]^**^	-22.052 [-31.905,-12.199]^***^	0.080 [0.040,0.120]^***^	-0.048 [-0.070,-0.027]^***^	-0.0002 [-0.0003,-0.0001]^***^
M2	-0.065 [-0.102,-0.028]^***^	-2.660 [-4.570,-0.749]^**^	-21.233 [-31.520,-10.947]^***^	0.078 [0.038,0.117]^***^	-0.046 [-0.068,-0.024]^***^	-0.0002 [-0.0003,-0.0001]^**^

Values are linear regression coefficients with 95% confidence intervals [in bracket] obtained from linear regression models

Crude model: Unadjusted

Model 1: Model adjusted for sex, age and BMI

Model 2: Model 1 + body fat (%), metabolic syndrome, smoking, physical activity, education and marital status

Sensitivity analyses were conducted to assess the robustness of the main findings (Table 4). When analyses were restricted to participants with BMI > 25 kg/m², each 1-point increase in MEDAS score remained significantly associated with lower NLR, PLR, SII and SIRI values, and with higher LMR. Comparable results were observed when limiting the sample to individuals presenting with obesity (BMI  30 kg/m²), as well as when analyses were restricted to participants with overweight or obesity combined with central adiposity and at least one metabolic complication. Finally, in a sensitivity analysis conducted on an expanded dataset that also included individuals with type 2 diabetes, cardiovascular disease, or a history of cancer within the previous five years, the observed associations remained materially unchanged. Overall, sensitivity analyses consistently confirmed the inverse relationship between adherence to the Mediterranean diet and systemic inflammatory indices.

**Table 4:** Sensitivity analysis

	*n*	NLR	PLR	SII	LMR	SIRI	MHR
Maintaining people with BMI > 25 kg/m^2^	1280	-0.057[-0.087,-0.028]^***^	-2.274[-3.820,-0.728]^**^	-18.117[-26.121,-10.113]^***^	0.0815[0.036,0.127]^***^	-0.041[-0.062,-0.020]^***^	-0.0002[-0.0003,-0.0000]^*^
Maintaining people with BMI ≥ 30 kg/m^2^	513	-0.054 [-0.091,-0.018]^**^	-2.753 [-4.944,-0.562]^**^	-17.570 [-28.872,-6.269]^**^	0.073 [0.008,0.139]^*^	-0.033 [-0.061,-0.005]^*^	-0.0002 [-0.0004,0.0001]
Maintaining people with BMI 30 kg/m^2^ or with BMI ≥ 25 kg/m^2^ + WtHR ≥ 0.5 + at least 1 metabolic complication	946	-0.054 [-0.082,-0.025]^***^	-3.012 [-4.720,-1.303]^**^	-19.254 [-27.738,-10.770]^***^	0.091 [0.038,0.144]^**^	-0.038 [-0.060,-0.015]^**^	-0.0002 [-0.0004,-0.0000]^*^
Including people with type 2 diabetes, cardiovascular disease or cancer in the past 5 years	2038	-0.060 [-0.093,-0.028]^***^	-2.357 [-4.043,-0.672]^**^	-19.690 [-28.711,-10.669]^***^	0.068 [0.030,0.107]^***^	-0.045 [-0.065,-0.025]^***^	-0.0002 [-0.0003,-0.0001]^**^

Values are linear regression coefficients and 95% confidence intervals obtained from linear regression models adjusted for sex, age, BMI, body fat, metabolic syndrome, smoking, physical activity, education, marital status

Abbreviations: NLR, neutrophil-to-lymphocyte ratio; PLR, platelet-to-lymphocyte ratio; SII, systemic immune-inflammation index; LMR, lymphocyte-to-monocyte ratio; SIRI, systemic inflammation response index; MHR, monocyte-to-HDL ratio

*p < 0.05, **p < 0.01, ***p > 0.001

## Discussion

In this large cross-sectional study conducted among adults seeking a weight loss dietary program, greater adherence to the Mediterranean diet was independently associated with a more favorable inflammatory profile, as reflected by lower NLR, PLR, SII, SIRI and MHR values and higher LMR values. These associations remained robust after adjustment for multiple potential confounders and were consistently confirmed across several sensitivity analyses. Specifically, comparable results were observed when analyses were restricted to individuals with overweight, to those presenting with obesity defined according to BMI criteria (≥ 30 kg/m²), as well as to those with excess body weight and central adiposity accompanied by at least one obesity-related comorbidity, defined as BMI ≥ 25 kg/m², waist-to-height ratio ≥ 0.5, and the presence of at least one metabolic complication. This finding is particularly relevant given that obesity is characterized by chronic low-grade inflammation arising from adipose tissue dysfunction and altered immune cell activity. The persistence of the associations in this subgroup suggests that greater adherence to the Mediterranean diet is associated with a more favorable inflammatory profile even among individuals at higher inflammatory and cardiometabolic risk. Furthermore, the inclusion of individuals with type 2 diabetes, cardiovascular disease, or a recent history of cancer did not materially alter the magnitude of the observed associations.

Although the magnitude of the associations observed for 1-point increase in MEDAS score was modest, the cumulative differences associated with larger variations in Mediterranean diet adherence may be more substantial. Specifically, each 1-point increase in Mediterranean diet adherence was associated with an approximate 3.4% reduction in NLR, a 1.9% reduction in PLR, a 4.6% reduction in SII, a 5.1% reduction in SIRI, and a 2.5% reduction in MHR relative to the median values observed in the overall study population. Conversely, LMR increased by approximately 2.0% per each additional MEDAS point. Taken together, these findings indicate that incremental differences in adherence to the Mediterranean diet were consistently associated with favorable changes across multiple complementary indices of low-grade systemic inflammation.

Several studies have investigated the relationship between overall dietary quality and composite inflammatory indices derived from routine blood tests. In a recent analysis of 3,442 cancer survivors enrolled in the United States National Health and Nutrition Examination Survey (NHANES), greater adherence to the Planetary Health Diet Index (PHDI) was inversely associated with both the SII and NLR [29], suggesting a link between healthier dietary patterns and lower systemic inflammation. Similarly, another NHANES-based study reported inverse associations between the Healthy Eating Index (HEI) and both NLR and SII [30]. In contrast, dietary patterns characterized by lower nutritional quality have been associated with a more adverse inflammatory profile. Specifically, adherence to a Western dietary pattern has been linked to higher NLR values [31], and higher consumption of ultra-processed foods has also been associated with increased NLR [32], further supporting the pro-inflammatory potential of poor-quality diets. Within this framework, the present study extends previous evidence by demonstrating that greater adherence to the Mediterranean diet is associated with lower levels of hematologic inflammatory indices in adults seeking a weight loss dietary program. These findings suggest that better overall dietary quality is associated with a more favorable inflammatory profile, with greater adherence to the Mediterranean diet showing consistent associations across multiple inflammatory indices.

The observed inverse associations between the adherence to the Mediterranean diet and hematologic inflammatory indices can be explained by several interconnected biological mechanisms. First, the Mediterranean diet is rich in plant-based foods, such as fruits, vegetables, legumes, whole grains, nuts, and olive oil, which provide bioactive compounds with anti-inflammatory and antioxidant properties. Among these, polyphenols may help reduce inflammation by modulating the NF-κB signaling pathway, a key regulator of inflammatory responses [33]. Activation of NF-κB stimulates the production of inflammatory molecules such as TNF-α, IL-6, and IL-1β. Modulation of this pathway by the phenolic compounds characteristic of the Mediterranean diet may therefore lead to lower production of these cytokines and, consequently, a reduction in systemic inflammation [33]. Second, the Mediterranean diet is characterized by the use of extra virgin olive oil as the primary source of fat and by a high consumption of fish and nuts, foods that are rich in monounsaturated fatty acids such as oleic acid and polyunsaturated fatty acids, particularly those of the omega-3 series. It is well established that omega-3 fatty acids exert significant anti-inflammatory effects through multiple biological mechanisms [34]. On the one hand, omega-3 help reduce the activation of intracellular pro-inflammatory pathways, including the NF-κB cascade [34, 35]. On the other hand, they modulate eicosanoid synthesis by decreasing the production of pro-inflammatory prostaglandins and leukotrienes derived from arachidonic acid and by promoting the generation of specialized pro-resolving lipid mediators such as resolvins, protectins, and maresins, which support the resolution of inflammation and the restoration of normal tissue function [34–36]. Third, the Mediterranean diet is characterized by a higher intake of dietary fiber, which not only improves glycemic control and insulin sensitivity but also promotes a healthier gut microbiota composition. This, in turn, enhances the production of short-chain fatty acids with anti-inflammatory properties, potentially helping to reduce low-grade inflammation [37, 38] by positively influencing inflammatory markers. Moreover, adherence to the Mediterranean diet has been consistently associated with lower adiposity and improved body fat distribution [39]. Given the central role of adipose tissue, particularly visceral fat, as an active source of pro-inflammatory cytokines [40], improvements in diet quality may indirectly reduce systemic inflammation by mitigating adipose tissue–derived inflammatory signaling. This pathway is especially relevant in populations with overweight and obesity, such as the present study sample.

Our findings suggest that greater adherence to the Mediterranean diet is associated with a more favorable inflammatory profile among adults with excess adiposity, supporting the need for further prospective and intervention studies. Given the established links between low-grade systemic inflammation and cardiometabolic health, future research should clarify whether the observed associations translate into clinically meaningful benefits.

This study has some limitations that should be acknowledged. First, the cross-sectional design precludes any causal inference. Second, MEDAS, like all food frequency questionnaires, relies on self-reported dietary intake and is therefore subject to recall bias. Moreover, being a short screener, it does not provide information on total energy or nutrient intake, preventing the exploration of potential confounding or mediation effects of specific dietary components. Third, although the inflammatory indices used are informative, they do not fully capture the complexity of immune response and systemic inflammation. Fourth, participants were recruited among individuals seeking a weight-loss dietary program, which limits the generalizability of our findings to the general population and calls for confirmation in more representative samples. Finally, as with any observational study, residual confounding cannot be entirely excluded.

Despite these limitations, this study has several strengths. To the best of our knowledge, this is the first study to investigate the relationship between adherence to the Mediterranean diet and inflammatory indices. The large sample size allowed for a precise estimation of effects, as reflected by the narrow confidence intervals. The use of multiple inflammatory indices further strengthens the robustness of our findings, as all consistently suggest a more favorable inflammatory profile associated with higher adherence to the Mediterranean diet. This robustness is further supported by the stability of results observed in sensitivity analyses. In addition, analyses were adjusted for body composition, a well-known determinant of systemic inflammation.

In conclusion, greater adherence to the Mediterranean diet was associated with a more favorable inflammatory profile in adults seeking a weight loss dietary program. These findings reinforce the role of dietary patterns as modulators of systemic inflammation and highlight the Mediterranean diet as a potentially valuable intervention in clinical nutrition settings. Future prospective studies and dietary intervention trials are warranted to confirm these observations and elucidate underlying mechanisms.

## Data Availability

The dataset analyzed during the current study is available from the corresponding author on reasonable request.
